# Priority trends and prospects of blackberry breeding in conditions of Central Russia

**DOI:** 10.18699/VJ20.641

**Published:** 2020-08

**Authors:** L.A. Gruner, B.B. Kornilov

**Affiliations:** Russian Research Institute of Fruit Crop Breeding, Zhilina, Orel district, Orel region, Russia; Russian Research Institute of Fruit Crop Breeding, Zhilina, Orel district, Orel region, Russia

**Keywords:** blackberries, breeding, trends and priorities of breeding, Central Russia, ежевика, селекция, направления и приоритеты селекции, средняя полоса России

## Abstract

This overview substantiates the possibility and expediency of blackberry breeding in Central Russia, where it is in demand, but not widespread in horticulture. Significant achievements of world breeding, which gave modern cultivars a large set of economically important qualities and growing interest in it all over the world, including Russian gardeners, make it relevant to work with blackberries as an object of selection, and as a promising garden plant. However, insufficient frost and winter hardiness of the bulk of the cultivars of this culture cause certain difficulties when growing it in the areas with cold winters to which the Central zone of Russia belongs. The expansion of the market of berry products also imposes increasingly high requirements on the complex of economic indicators of new cultivars, primarily the quality of blackberry fruit. In this regard, improving the existing range of varieties of the culture, increasing its adaptive properties and commodity qualities of berries are urgent tasks for breeders when creating new cultivars. The relevance of blackberry breeding is also dictated by the fact that in Russia its domestic range of varieties is represented by only one modern cultivar obtained in the southern region and adapted, first of all, to it. For the Central zone of the country, the cultivars of this plant have not been developed (except for the limited experiments of I.V. Michurin conducted almost 100 years ago). Therefore, the breeding of adapted cultivars of the culture in the climatic conditions of this region may be promising. It is also possible to grow here (with shelter for the winter) the cultivars already created abroad that can give with the right agricultural technology a good industrial harvest, which is confirmed by the practice of amateur and farm gardening, as well as scientific research. The purpose of this work is to designate the leading directions of blackberry breeding, the most important in the conditions of Central Russia and to show prospects of the development of new cultivars of this valuable culture in the specified climatic zone. The analysis of world trends and experience in the blackberry breeding and variety study, as well as the results of our own research of the culture conducted in the Orel region, allow us to consider it promising and relevant to work on improving the range of varieties of this plant in Central Russia. All priority areas of blackberry breeding, indicated in foreign and domestic breeding programs (winter hardiness, high quality of fresh and processed fruit, the correct shape of berries, their large size, the necessary values of biochemical composition, high productivity of plants, thornless shoots and high resistance to diseases and pests), are relevant for this region of our country, while high winter hardiness is currently the most important of them.

## Abstract (ru)

В обзоре обосновывается возможность и целесообразность селекции ежевики в центральной части России, где она является востребованной, но малораспространенной в садоводстве ягодной куль- турой. Значительные достижения мировой селекции, давшие современным сортам большой набор хозяй- ственно важных качеств, растущий интерес к культуре во всем мире, в том числе у российских садоводов, делают актуальной работу с ежевикой и как с объектом селекции, и как с перспективным садовым рас- тением. Однако недостаточные морозо- и зимостойкость основной массы сортов этой культуры создают определенные трудности при выращивании ее в зонах с холодными зимами, к которым относится средняя полоса России. Расширение рынка ягодной продукции тоже предъявляет все более высокие требования к комплексу хозяйственных показателей новых сортов, в первую очередь к качеству плодов ежевики. В свя- зи с этим улучшение имеющегося сортимента культуры, повышение его адаптивных свойств и товарных качеств ягод – насущные задачи для селекционеров при создании новых сортов. Актуальность селекции диктуется также тем, что в России отечественный сортимент ежевики представлен всего одним сортом – Агатовая, полученным в южном регионе и адаптированным прежде всего к нему. Для центральной же зоны страны сорта не создавались (за исключением ограниченных опытов И.В. Мичурина, проведенных около ста лет назад). Поэтому выведение адаптированных сортов ежевики в климатических условиях этого региона может оказаться перспективным. Не исключается также возможность выращивания здесь (при укрытии на зиму) уже созданных за рубежом сортов, которые могут давать при правильной агротехнике хороший про- мышленный урожай, что подтверждает практика любительского и фермерского садоводства, а также вы- полненные научные исследования. Цель данной работы – на основании анализа результатов зарубежных и отечественных исследований обозначить ведущие направления селекции ежевики, важнейшие в условиях средней полосы России, показать перспективность создания новых сортов этой ценной культуры в указан- ной климатической зоне. Анализ мировых тенденций и опыта в селекции и сортоизучении ежевики, а также результаты собственных исследований культуры, проведенных в условиях Орловской области, позволя- ют считать перспективной и целесообразной работу по совершенствованию сортимента этого растения в средней полосе России. Все приоритетные направления селекции ежевики, обозначенные в зарубежных и отечественных селекционных программах (зимостойкость, высокое качество плодов в свежем и перерабо- танном виде, правильная форма ягод, крупный их размер, необходимые значения биохимического состава, высокая продуктивность растений, бесшипность побегов, высокая устойчивость к болезням и вредителям), актуальны и для данного региона нашей страны; при этом важнейшим направлением является в настоящее время создание сортов с высокой зимостойкостью.

## Introduction

In recent years, more and more attention has been paid to berry crops that are not widely distributed in Russia, such as actinidia, blueberries, blackberries, honeysuckle, viburnum, sea buckthorn and others, which can significantly expand the range of berry products and enrich the diet of the population with useful substances, while representing considerable commercial interest. Their fruits are a rich source of valuable substances for the human body (microand macronutrients, vitamins), including antioxidant action. The content of these substances in them is often higher than that of traditionally grown in most regions – strawberries, raspberries, black currants, etc., or is at the same level (Sedov, Gruner, 2014), which confirms the importance of actively involving cultivars of such plants in the practice of gardening.

Among the crops of this group, blackberries are of considerable interest at present, which may eventually occupy a worthy niche in Russian berry growing, as has happened in a number of countries around the world (Strik et al., 2008; Strik, Finn, 2012), where it is among the leading berry plants (Clark, Finn, 2011, 2014; Finn, Clark, 2012).

Blackberries bear fruit after most other berry crops, significantly extending the pipeline of vitamin products in the growing regions. Its fruits contain a significant amount of important biologically active substances of the antioxidant complex (Gruner, Anikeyenko, 1995; Connor et al., 2005; Kolbas et al., 2012; Milošević et al., 2012; Lee, 2017), which are involved in many processes of human metabolism (Kolbas et al., 2012). Their number is: from 500 to 900 mg/100 g of P-active substances-flavonoids (including 17 to 30 mg/100 g of ellagic acid and 85–390 mg/100 g of ellagotanins), from 10 to 50 mg/100 g of ascorbic acid, about 0.6 mg/100 g of carotenoids. In addition, blackberries contain 5 to 14 % sugars (mainly glucose and fructose), 1.3 % organic acids, as well as a significant number of important mineral macro- and microelements (in terms of dry weight) – phosphorus (up to 254 mg/100 g), calcium (about 283 mg/100 g), magnesium (up to 315 mg/100 g), iron (up to 11 mg/100 g), etc. Pleasant taste and delicate aroma of the large black fruit in combination with the specified components of the chemical composition make this culture attractive to gardeners and numerous berry consumers in different regions of our country. The demand for and at the same time the shortage of blackberries in the market of garden products in Russia, including the middle zone, is indirectly evidenced by the high prices for its fruits and planting material of cultivars.

Improved over the years of breeding productivity indicators (up to 20 t/ha) (Clark et al., 2019), high self-fertility (Gruner, 2019) and resistance of many modern cultivars to the most dangerous diseases and pests (Clark, Finn, 2011; Finn, Clark, 2012), good recovery ability after various injuries (Gruner, 2019), relative ease of vegetative reproduction (Podorozhny, Romanova, 2010; Knyazev et al., 2012), responsiveness to bush formation (Clark, Finn, 2011; Takeda et al., 2013), new thorn-free cultivars allow growing blackberries at present without any problems. A certain vulnerability of this plastic garden crop is associated with reduced winter hardiness of almost all its cultivars in the regions with cold and long winters, especially with unstable snow cover, as in the middle zone of Russia (Yakimov, 2010; Evdokimenko, Kulagina, 2015; Gruner, 2019). Therefore, the introduction of blackberries in these climatic conditions is associated with corresponding difficulties, which can be overcome in two ways: selection and technological, as well as their combination.

Blackberry breeding in the world has passed a long historical path (Darrow, 1937; Shoemaker, 1958; Ourecky, 1981; Vitkovskiy, 2003; Clark, Finn, 2011; Finn, Clark, 2012), which resulted in numerous (several hundred) highly productive cultivars (Ourecky, 1981; Yakimov, 2010; Clark et al., 2012; Finn, Clark, 2012). Modern blackberry assortment with a complex of valuable properties allows using it as a source material for further selection, improving individual qualities, or adding additional ones without losing the rest. This makes plant breeding promising, including in the conditions of the center of our country, where there are not as many competing crops that produce high-quality fruits as in the South. Encouraging in terms of promoting blackberries to the North are the positive results already obtained on its selection for winter hardiness in the United States and some European countries (Danek, Kolodziejczak, 1993; Stanisavljevic, 1999; Danek, Orzeł, 2004; Clark et al., 2012; Clark, 2013; Orzeł et al., 2016).

Orel region, where this work is carried out, has a combination of climatic factors of the growing season (Agroclimatic Reference Book…, 1960), in most cases favorable for the growth, development and fruiting of blackberries (Gruner, 2019). The success of breeding blackberries, as well as other fruit and berry crops, in any climate zone is determined, first of all, by the availability of good source material and the correct choice of priority areas, which should be based on the already achieved world results and correspond to them.

## 1 Taxonomic affiliation, the most important species for breeding and some morphobiological features of blackberries

Blackberries are included in the genus Rubus L., subgenus Eubatus Focke (= Rubus Watson), which, according to various authors, has 132 species in the world’s flora (Focke, 1910) to 200 or more species (Rozanova, 1937; Vitkovskiy, 2003) and a significant number of interspecies forms. The complexity of the systematics of wild blackberries was mentioned by the famous researcher of this plant, S.V. Yuzepchuk (1941). Within this subgenus, natural polyploidy is widespread, represented by a polyploid series of forms that have sets of chromosomes from 2x (2n = 14) to 12x (2n = 84) with the main number x = 7, including aneuploids of different ploidy levels. At the same time, such series can also be within some species (Rozanova, 1937; Ourecky, 1981; Clark, Finn, 2011). The size of blackberry chromosomes is 1–4 μm (Clark, Finn, 2011). In its cultivated cultivars, polyploid rows also occur, with a predominance of tetraploids (Thompson, 1995; Clark, Finn, 2011).

Wild species of North America and Europe became the originators of most cultivars of the Eubatus subgenus (Rozanova, 1937; Vitkovskiy, 2003). The most significant in the creation of blackberry cultivars were such North American species as: R. allegheniensis Porter, R. argutus Link. and R. canadensis L. (which gave rise to the first erected and most hardy cultivars, such as Agawam, Jumbo, Lawton, Snyder, Erie, etc.), R. ursinus Cham. & Schlecht., R. macropetalus Dougl., and R. loganobaccus Bailey (the most important species from which the best trailing cultivars of blackberries and raspberry-blackberry hybrids have been obtained: ‘Logan’, ‘Young’, ‘Boysen’, etc.), R. laciniatus Willd. (well-known ‘Thornless Evergreen’ was obtained from its thorn-free chimera), R. trivialis L. (one of the parent forms of the erected drought-resistant cultivar Brazos, which was actively used in creating modern cultivars), R. ulmifolius Schott. (a tetraploid source of the recessive thornless gene, ‘Merton Thornless’ was derived from it), (Darrow, 1937; Clark, Finn, 2011), and others. It is usually impossible or very difficult to determine the species identity of modern cultivars, since their genomes usually contain the genoplasm of several species and their hybrid descendants, as well as cultivars of different origin, which in turn is complicated by polyploidy and related heterozygosity of the culture (Rozanova, 1937; Ourecky, 1981; Clark, Finn, 2011).

Blackberry flowers are usually bisexual, but there are also dioecious species (for example, R. ursinus in the United States). Self-pollination is good, but cross-pollination is also successful. The color of the corolla varies from pure white to bright pink. The stamens and pistils are numerous. The fruit is a compound drupe, with a color from dark cherry or blue to almost black, sometimes with a glaucous coating, when maturing, separating from the calyx along with the edible fruit. The number of drupes in it can vary from a few pieces in wild species and forms (for example, R. caesius L.) to one and a half hundred in modern cultivars (as in ‘Natchez’). Set of drupes is usually high, often provided by facultative apomixis (pseudogamy), characteristic of polyploid forms of the Eubatus subgenus in various types of pollination (Ourecky, 1981). Flowers and fruits are collected in racemes containing them from several pieces to several dozen.

In Russia, the largest number of wild species is distributed in the Caucasus (about 40), but in the conditions of the middle zone of our country, only two species prevail: R. caesius L. with trailing shoots and R. nessensis W. Hall with erected canes (Yuzepchuk, 1941; Grossgeim, 1952).

The blackberry plant is a semi-shrub with a long-term underground part (branched rhizome with branching roots and buds from which shoots grow) and above-ground, consisting of two types of stems: generative biennial and vegetative shoots of the current year (annual). After the crop matures, the stems of the second year of life die off. There are also primocane forms that lay generative organs and bear fruit on the shoots of the current year.

There are 3–4 main morphological groups of blackberries that differ in the nature of growth and way of natural vegetative reproduction (Finn, Strik, 2014; Gruner, 2014). The group of erected blackberries includes cultivars and forms that have vertically growing shoots and restrained early ending growth (until the beginning of August in the middle zone of Russia). The natural way of vegetative reproduction in them is with root offshoots (as in red raspberries R. idaeus L.). Trailing blackberry plants have a protracted growth, long (up to 3–8 m) shoots of various thickness, which by the end of the growing season slow down the growth, but do not stop, and in the upper zone form a section of rhizogenesis with reduced leaves, rooting under favorable conditions. Representatives of semi-erect and semi-trailing blackberries complete or significantly slow down the growth after erect, but earlier trailing forms (in the middle zone of Russia, it is the beginning of September). Given that the last two groups are similar to each other, in foreign literature they are usually combined, calling them “semierect” i. e. semi-straight growing (Finn, Strik, 2014).

## 2 State of blackberry breeding in Russia

In our country, blackberry breeding is now at the initial stage of its path, despite the abundance of wild species and forms, especially in the southern regions, where they could have long served as a source material for selection, but aroused interest mainly as botanical resources (Yuzepchuk, 1941; Grossgeim, 1952). At the beginning of the last century, this plant, as an object of selection, seriously drew the attention of a well-known breeder and experienced gardener – I.V. Michurin (1949), who, as a result of sowing seeds from free pollination of American cultivars, selected several promising seedlings, which later became cultivars and received recognition in amateur gardening. But in the future, these cultivars were preserved mainly in the collections of scientific institutions, as the plants had thorned shoots, low winter hardiness and were affected by diseases (‘Izobilnaya’, Tekhas).

Major domestic works on blackberries and their cultivars, created by that time in the world, were published in 1930s (Bologovskaya, 1934; Rozanova, 1937). Later, translated foreign publications with detailed information about this culture were published (Shoemaker, 1958; Ourecky, 1981; etc.). Collections of wild species and some foreign cultivars in the 1950s–70s and subsequent years were collected at Maikop (main collection) and Pavlovskaya (single samples) experimental stations of VIR (N.I. Vavilov All-Russian Institute of Plant Genetic Resources) in order to study and select the most promising of them for breeding. N.L. Neronova (1973), a participant of several scientific expeditions to the Caucasus in order to replenish the species collection of blackberries of MOS VIR (Maikop Experimental Station of the N.I. Vavilov All-Russian Institute of Plant Genetic Resources), wrote about the great potential value of wild Caucasian blackberry species, including for introduction into culture in this zone.

Several works performed in the southern zone of horticulture at the end of the last and beginning of this century (Gruner, 1992; Semyonova, Dobrenkov, 2001; Zakharova, 2002) using the gene pool of the Maikop OS VIR culture were devoted to the study of the biological characteristics and economic value of blackberries and the selection of genetic sources for breeding. By this time, a collection of blackberries has been also formed on the Crimean OSS VIR (Crimean Experimental Selection Station of the N.I. Vavilov All-Russian Institute of Plant Genetic Resources), where targeted selection of this culture (the collection gene pool of which numbered 25 introduced cultivars during the study period) has been carried out since 2003 (Podorozhny, 2016), culminating in 2016 with the inclusion of the first and only modern domestic cultivar – ‘Agatovaya’ in the State Register of the Russian Federation (State Register…, 2019). The priority areas of blackberry breeding in this institution are both improving the indicators of adaptability to climate factors in the southern region (including drought and heat resistance), and improving other characteristics identified as leading in domestic and foreign breeding programs, such as berry quality, productivity, thornlessness, etc. (Kichina et al., 1995; Clark, Finn, 2011; Podorozhny, 2016).

The most obvious reasons for the long absence of breeding research on blackberries in Russia were, first, the abundance of other valuable berry crops distributed in different regions and adapted to them, second, a large variety of wild-growing species of this plant in the southern climate zone, which gave the local population a sufficient number of berries, and third, the strong thornness of intensively growing shoots, both in wild-growing species and in old foreign cultivars, which made blackberries perceived more as a weed and inconvenient for cultivation, and, in-fourth – low winter hardiness of its aboveground part, which did not allow to count on stable berry yields.

With the introduction of a whole series of modern foreign blackberry cultivars to our country over the past decade and a half, the situation in crop breeding may change significantly, since the level of most economic indicators is very high and makes it possible to choose the best genotypes for crosses. Collections of the latest generations of foreign blackberry cultivars are already available and are being studied in a number of Research Institutes, including in the Central part of Russia: VNIISPK (Russian Research Institute of Fruit Crop Breeding, Orel region) – 28 cultivars, Bryansk SAU – about 10 cultivars (private message of Evdokimenko S.N.), I.V. Michurin Federal Research Center (Tambov region) – about 30 cultivars (private message of Gurieva I.V.).

## 3 Blackberry breeding abroad, priorities and achievements

For more than 170 years, blackberries have been selected in the United States (Darrow, 1937; Shoemaker, 1958; Ourecky, 1981; Vitkovskiy, 2003; Clark, Finn, 2011; Finn, Clark, 2012), where a large variety of wild species of this plant, which gave rise to many cultivars, is also concentrated. Some of the first cultivars appeared spontaneously and were accidentally discovered in private gardens and wild populations, serving as a base and incentive for subsequent selection search (Darrow, 1937; Clark, Finn, 2011). A big breakthrough in blackberry breeding was the development of the thornless ‘Merton Thornless’ cultivar in England (John Innes Horticultural Institute program), from which the recessive gene of thornlessness was transmitted to many modern tetraploid (2n = 28) cultivars (erect and semi-erect). Two other donors of genes of dominant thornlessness, S_f_ and S_fl_, ‘Austin Thornless’ (2n = 56) and ‘Lincoln Logan’ (2n = 42) were used in the creation of trailing thornless cultivars (for more information, see section 5.4).

To date, US scientists are recognized as world leaders in creating and improving the assortment of blackberries. Their research gave an impetus to the development of culture breeding in other countries. The main contribution to the creation of new cultivars of this plant was made by scientists of the world’s leading breeding institutions, such as: USDA-ARS Beltsville St. Maryland (Dr. D. Scott, Dr. D.P. Ink) – USA; Arkansas University (Dr. J. Moore, Dr. J. Clark) – USA; United States Department of Agriculture ARS St. Oregon (Dr. Ch. Finn, Dr. B. Strik) – USA; Scottish Crops Research Institute (Dr. D. Jennings) – Great Britain; Fruit Experiment Station of the Research Institute of Pomology and Floriculture (Dr. J. Danek, Dr. A. Orzel) – Poland; New Zealand Institute for Plant and Food Research (Dr. H. Hall) – New Zealand; Fruit Research Institute, Čačak (Dr. M. Stanisavljevič) – Serbia and others

All modern breeding programs are aimed at further improving the assortment of blackberries by leading economic characteristics. The analysis of major reviews on blackberry breeding (Ourecky, 1981; Clark, Finn, 2008, 2011; Finn, Clark, 2012) shows that the priority directions in the world breeding programs are currently: the quality and size of fruits, productivity, plant adaptability to climate factors, thornlessness, optimal architecture of shoots, fruiting on canes of the current year, resistance to diseases and pests. In other words, the range of problems to be solved remains quite wide, since the requirements for cultivars are constantly increasing, while the genetic potential of the crop identified by many years of research (Clark, Finn, 2011; Finn, Clark, 2012) allows to count on the success of their solution.

It should be noted that the first place is given to the quality of fruits associated with increased market requirements. The emphasis in the selection of adaptability to low winter temperatures in the United States, where for a long time there was active selection for this trait in combination with the straightness of shoots, partly shifted to another valuable direction, also in the future able to largely solve the problem of winter hardiness – the creation of primocane cultivars of blackberries (Clark, Finn, 2011; Clark et al., 2012; Finn, Clark, 2012; Clark, 2013, 2014) (for more information, see section 5.2).

The main results of the long-term and tedious work on the blackberry breeding were high-yielding cultivars with a complex of valuable characteristics. New breeding achievements have aroused great interest in the culture around the world, the level of production of its berries has grown significantly over the past decades and continues to grow (Strike et al., 2008; Strike, Finn, 2012; Clark, Finn, 2014).

The breeders managed to increase the winter hardiness in combination with the plant thornlessness only to a certain level (Stanisavljevic, 1999; Clark, Finn, 2011; Orzeł, 2016; Telepenko, 2018), which is still insufficient for regions with long frosty periods in winter, such as those in the middle zone of Russia (Evdokimenko, Kulagina, 2015; Gruner, 2019). Therefore, along with the appearance of industrial cultivars, the best climatic zones for growing blackberries were determined, and technologies for its cultivation began to be worked out, including those that provide winter shelter for shoots (Takeda, Handley, 2006; Takeda et al., 2013; Mettler, Hatterman-Valenti, 2018). As a result, the leading producers of blackberries in the world today are the United States, Mexico, China, Serbia, Hungary, New Zealand, and in general – about 30 countries (Strik et al., 2008), where climate conditions allow it.

## 4 Factors limiting blackberry cultivation in the Central part of Russia (on the example of the Orel region)

Orel region, where our blackberry research is conducted, is located in a zone of moderate continental climate, generally favorable for gardening (Agroclimatic Reference Book…, 1960). The temperature of the coldest month (January) is –9…–10 °C. The absolute minimum air temperature for the long-term period is –39.9 °C in the region (2012, according to the VNIISPK weather station). According to the average annual data, frosts stop in the region in the second five days of May (possible fluctuations in the timing of frosts – from the first decade of April to the first decade of June). The average dates of autumn frosts are at the end of September (the earliest start of frosts was observed in the first decade of September, and the latest was in the third decade of October). The snow cover reaches maximum height from mid-February to mid-March. Its average height is 20–25 cm.

During our research, according to VNIISPK meteorological observations, significant decreases in winter temperatures (corresponding to regional minimums) were observed in late November 2014 (–20 °C), in mid-December 2016 (–20.6 °C), in the first decade of January 2015 (–24.5 °C), in the second decade of January 2016 (–29.3 °C), in early February 2017 (–31.5 °C), in late February (–26.0 °C) and at the end of the third decade of March 2018 (–21.6 °C). At the same time, in January, February and March in some years there were long (up to two or more weeks) periods of round-the-clock negative temperatures (with lows at –10…–15 °C) (Gruner, 2019).

The limit of frost resistance of the main mass of blackberry cultivars are considered –10…–20 °C, and only for some of them –25…–30 °C (Gruner, 1986; Takeda, Handley, 2006; Wojcik-Seliga, Wojcik-Gront, 2013; Telepenko, 2018, etc.). It is established both experimentally, using artificial freezing of cuttings, and by field observations, which are the most accepted when evaluating breeding seedlings (Kichina et al., 1995; Kazakov et al., 1999; Clark, Finn, 2011). In any case, the specified limits of blackberry resistance to frost are not sufficient for the region of our research, and this is the main obstacle to the introduction of the crop in this climate zone. Nevertheless, there is already a positive experience of fairly large blackberry plantations (4–7 ha) in the South of the Central zone of Russia – in the Voronezh region: farm “Sladunika” (URL: https://sladunika.ru) and “Divny Sad” (URL: https://divnyi-sad.ru). However, even there they usually require winter shelter, with the exception of certain cultivars that are not frozen even in the harshest winters, such as the Polish cultivar ‘Orkan’ and the old American cultivar ‘Agawam’ (private message of the head of the farm “Divny Sad” – A.N. Prodan).

In addition to the lack of winter hardiness of the main assortment, the factors that hinder the cultivation of blackberries in the middle zone of the country and other regions with cold winters (–20 °C and below), can be attributed to the related need for shelter of plants (a labor-intensive and costly event), as well as the diversity of cultivar habit, requiring an individual approach to each of them when growing; mandatory use of trellis due to high productivity and long shoots; and sometimes insufficient knowledge of the biology of the plant, resulting in certain errors in agrotechnics.

## 5 Results of blackberry cultivar study in the center of Russia, current breeding trends, genetic sources of valuable traits

Purposeful selection evaluation of cultivated blackberries in the climatic conditions of Central Russia has not been carried out since the creation of the first cultivars by I.V. Michurin (Michurin, 1949). In connection with the increased interest of the population in cultivars of this plant, their biology and cultivation techniques, the appearance of new selection products at the market, we conducted research on the variety study of blackberries in experimental plantings of the Department of berry breeding of VNIISPK in 2014–2018 (Orel, Russia). To evaluate the collection and selection material, we used generally accepted research methods (Kichina et al., 1995; Kazakov et al., 1999).

The cycle of works performed in these years to study the adaptive and other economic qualities of representatives of the main morphological groups of blackberries (erect, trailing and intermediate between them) has shown that this valuable berry crop can be successfully grown here under certain conditions (first of all using winter shelter) and give high yields of berries. This requires trellis and variety-specific preparation of plants for winter (Gruner et al., 2018). It is important to select cultivars according to the maturation period, since adverse weather conditions during the growing season of individual years can lead to non-maturation of part of the crop of the most late cultivars, prolonged growth of shoots of the current year and, as a result, their significant freezing in winter (Gruner, 2019). It has been found that, as a rule, all blackberry phenophases occur at a favorable time for the culture and fit into the growing season of the region (Gruner, Kuleshova, 2014). It is revealed that among the studied and involved in the collection new blackberry genotypes (for the latest information prior) there are cultivars (‘Agawam’, ‘Erie’, ‘Thornfree’, ‘Brzezina’, ‘Ouachita’, ‘Loch Tay’, ‘Natchez’, ‘Chester’, etc.) and selected forms (seedlings of ‘Cheyenne’, ‘Black Satin’, ‘Loch Ness’, and hybrid form ‘Thornfree’ × R. caucasicus I, etc.) that allow to count on successful selection for improvement as indicators of adaptability, i. e. hardiness and drought resistance (Gruner, 2019) and other economically important traits, including weight, taste and other qualities of the berries (Clark, Moore, 2005, 2008; Yakimov, 2010).

Practical tips and positive forecasts for growing blackberries in the more severe conditions of the Samara region, information about the numerous cultivars of the world assortment of this culture were reflected in the book written by V.V. Yakimov, a connoisseur of this culture (Yakimov, 2010), based on his own experience and a review of foreign literature sources.

The authors of the study of blackberry winter hardiness without winter shelter in the Bryansk region rightly believe that it is impractical to grow blackberries in this zone without such shelter due to the freezing of most cultivars to the level of snow, but some of the cultivars studied by them stand out (‘Thornfree’,’ Black Satin’, ‘Gazda’, ‘Orkan’, ‘Marion’ and ‘Smoothstem’) as promising for household and industrial use in the region (Evdokimenko, Kulagina, 2015). Current trends in blackberry breeding in Russia were formulated earlier (Kichina et al., 1995) and supplemented with respect to the Central zone in this article. Of these, in the first place is breeding for winter hardiness and necessary qualities of blackberry plants associated with it. Let’s focus on this direction and some others in more detail.

**5.1 Breeding for winter hardiness**

The creation of hardy blackberry cultivars was important for the United States due to the significant freezing of most Eubatus representatives in almost all regions of this country (Finn, Clark, 2012). The work of North American scientists to create erect genotypes originally obtained from wild species with such a habit – R. allegheniensis, R. argutus and others was valuable in the breeding of relatively frost- and winter-hardy blackberry cultivars. The first cultivars with the genoplasm of these species were thorny, characterizing by increased frost resistance and restrained growth: ‘Agawam’, ‘Lawton’, ‘Snyder’, ‘Erie’, ‘Darrow’, and others (Darrow, 1937; Clark, Finn, 2011). Winter hardiness of such cultivars is associated with the early completion of growth processes; they are also characterized by the depth and duration of organic dormancy. As a result of the long-term breeding, thorny erect ‘Illini Hardy’ and thornless semi-trailing ‘Chester Thornless’ are among the most winter-hardy cultivars (Yakimov, 2010; Finn, Clark, 2012). However, over time, US breeders have recognized that climatic conditions with regular winter temperature drops to certain critical values are necessary for effective selection of genotypes with higher winter hardiness (Clark, Finn, 2011; Finn, Clark, 2012).

The latest generations of erect blackberries (‘Navaho’, ‘Arapaho’, ‘Natchez’, ‘Ouachita’, ‘Caddo’, and others, received at Arkansas University, USA) (Andersen, 2001; Clark, Moore, 2005, 2008; Clark, Finn, 2011; Clark et al., 2019), have thornless canes and improved winter hardiness, however, they complete growth (our preliminary observations) later than thorny ancestors, which is probably connected with the involvement of thornless, but long-term vegetative genotypes and therefore moderately winter-hardy genotypes (in particular, ‘Thornfree’). Therefore, in the Central zone of Russia, the winter hardiness of these cultivars is likely to be insufficient and closer to this indicator in semi-trailing forms. The morphological proximity of new erect cultivars to this intermediate group is also confirmed by their creators (Finn, Clark, 2012). At the same time, crossing such cultivars among themselves can probably lead to a certain increase in the hardiness of hybrids, due to the saturation with the genoplasm of the hardy ancestors of their parents, in particular, ‘Darrow’, which was used in the creation of most of them (Clark, Moore, 2005, 2008; Clark et al., 2019). Among the blackberry cultivars, checked by us in the Central zone of Russia in winter, without shelter, only the old American erect tetraploid (2n = 28) cultivar ‘Agawam’ can be grown here, however, when using it in breeding, one should take into account the dominant thornness of shoots and the formation of a large number of root offshoots, which are absent in modern thornfree cultivars with erect habit.

Breeding for increased winter hardiness of Eubatus representatives is successfully carried out in Poland (Fruit Experiment Station of the Research Institute of Pomology and Floriculture), where a number of cultivars were obtained that do not require shelter in the climatic conditions of this country (Danek, Kolodziejczak, 1993; Orzeł et al., 2016) and which, as already mentioned, in the South of Central Russia show good resistance to frost. This is, first of all, the mentioned cultivar ‘Orkan’, which confirmed the specified quality also in the Ukraine along with the American cultivar ‘Ouachita’ (Telepenko, 2018). These cultivars can be promising in the course of further breeding improvement of blackberry winter hardiness indicators, combined with the thornlessness of shoots.

For cover cultivars in the Central zone of Russia, increased winter hardiness and elasticity (flexibility) of shoots are also relevant, necessary for significant snow cover, garter to the trellis, shelter for the winter; trailing and semi-trailing habit of the bush and early ripeness. According to our research (Gruner, 2019) and preliminary observations, ‘Loch Tay’ (from the VNIISPK collection) and the hybrid form ‘Thornfree’ × R. caucasicus I, as well as seedlings LN-4, 13, 14 obtained from seeds from open pollination of ‘Loch Ness’ can be potential sources of these traits’ genes. In the conditions of the region, it may be useful

to study also cultivars with a typically trailing habit (at least for the purposes of amateur gardening), which are laid on the ground for winter shelter easier than others, giving preference to thorn-free genotypes (with genes S_f_ and _Sfl_).

**5.2 Breeding of primocanes fruiting**

One of the current trends that can solve the problem of winter hardiness of blackberries in the future is the creation of new primocane cultivars. Fruiting on shoots of the current year is a relatively new direction in blackberry breeding, but it is very important for expanding the production of berries both to the South and to the North (Clark, Finn, 2011). In the southern regions with warm winters, such cultivars fully bear fruit (and can even produce two crops per season) without the impact of negative temperatures, which ordinary (non-primocane) cultivars need, in particular, for normal differentiation of generative buds. When moving blackberries to the North, shoots of primocane cultivars, in the case of early (before the onset of negative temperatures) ripening of berries on an annual wood, can be cut (or mowed) after harvest, thus solving the problem of winter hardiness and resistance to diseases and pests.

The original source of the gene for this trait was discovered in 1949 in a natural population of erect diploid blackberry form (2n = 2x = 14), called ‘Hillquist’ after the name of the person who found it (L.G. Hillquist from Ashland) in the United States. From it, by breeding (crossing with the tetraploid cultivar ‘Brazos’ at the University of Arkansas), tetraploids were obtained (including the form Ark. 593, used in further selection), bearing the primocane trait. The recessive nature of the gene for this trait is assumed (Clark, 2008; Clark, Finn, 2011). ‘Prime-Jan’ and ‘Prime Jim’ (2003 and 2004) were the first breeding achievements of this type, Prime-Ark-45 (2009) and subsequent cultivars have improved fruit quality. The most important results in this direction were obtained at the University of Arkansas in the USA (where work on the creation of such cultivars continues). Breeding for primocanes is also conducted in Poland (Orzeł et al., 2016). In the currently created representatives of cultivars of this type of fruiting, berries can fully ripen on the shoots of the current year only in the southern zones of horticulture. Therefore, the goal of breeding in this direction, including in the center of Russia, is to provide earlier full ripening on one-year shoots and greater winter hardiness of the underground parts of plants. All other parameters of cultivars and breeding directions in the Central zone of our country correspond to the world priorities identified by leading breeders based on the longterm experience with blackberries (Clark, Finn, 2008, 2011; Finn, Clark, 2012). We give them below.

**5.3 Breeding for improved fruit quality**

Most modern blackberry cultivars have a set of indicators of berries that ensure their demand in the market and were formed by long-term breeding. This also applies to representatives of the group of erect and semi-erect cultivars, which have received a lot of attention to improving the quality of berries (Clark, Moore, 2005, 2008; Clark, Finn, 2011).

The sweet taste of fruit (provided by the amount of soluble solids, SS = 10–15 %) is considered key to expanding the market for fresh blackberry products (Clark, Finn, 2011). In addition to this indicator, acidity, aroma, and tartness are also important. Other traditional high-quality components are the density of the berry, the strength of the skin, small seeds, bright color, easy separation during harvesting, and good post-harvest safety.

When creating new cultivars, it should be taken into account that the ripening of berries is a special period when growing blackberries. On the one hand, the fruits acquire a cultivar-specific taste, gain the maximum amount of sugars, acquire juiciness and aroma, on the other hand, their transportability decreases, and a beautiful gloss is often lost. For this reason, berries are often removed at the stage of technical maturity, and they are not suitable for dessert consumption. The task of breeders is to combine high taste qualities in mature fruits (first of all, sweet taste) with the brilliance of black drupelets and the strength of the skin for successful transportation.

Post-harvest characteristics of blackberry berries include: attractiveness of appearance (preservation of black color, gloss), strength of the fruit (evaluated before and after storage), good taste, lack of the development of fungal and bacterial infections. The quality of berries after harvesting strongly depends on weather conditions during harvest, so it is practiced to grow blackberries, as well as other berry crops, in high tunnels (Finn, Clark, 2011; http://www.korolevagro.ru/teplichnye-tekhnologii/vysokie_tunneli/).

For processing, blackberries must have an intense aromatic taste and color, a high level of soluble solids and titrated acidity, low pH and low drupeletness. These characteristics must be preserved when the berries are frozen. There are significant differences between the cultivars of this crop in the content of phenolic compounds of the antioxidant complex (Connor et al., 2005), which allows for selection to improve this indicator in the offspring.

The shape of blackberry fruit can vary significantly (Gruner, 2014), which means that there are prospects for its selective improvement. It is important to equalize the shape, the fruit are not acceptable cloven or invalid. Desirable berries are rounded, conical or barrel-shaped (cylindrical). It is believed that long berries can provide the most attractive appearance of fresh fruit (Clark, Finn, 2011).

Large size of berries is one of the main tasks of blackberry breeding, and is a component of productivity in addition to the marketability indicator. However, it is considered that an excessive mass of berries (more than 10–15 g) is undesirable for processing and placement in packages, as well as for berry mixes, where such fruits will prevail. Large fruits are varieties of ‘Natchez’, ‘Triple Crown’, ‘Kiowa’, ‘Tupi’ and a number of other cultivars have large size of fruit (Yakimov, 2010), which are potential sources of genes for this trait. The weight of the berry may vary slightly within the cultivar depending on the conditions and methods of cultivation.

The productivity (yield per bush) of blackberries is usually high, but varies greatly both between cultivars and within a single cultivar, depending on climatic conditions and growing technology, and can reach 20 or more kilograms. However, an excessive crop can lead to a decrease in shoot-forming ability (Clark, Finn, 2011) and even in the frequency of fruiting. Therefore, it is necessary to manage the productivity of high-yielding cultivars with the help of timely formation of shoots, their normalization. Sources of high productivity genes are ‘Thornfree’, ‘Chester Thornless’, ‘Black Satin’, ‘Loch Ness’, ‘Triple Crown’, and many others (Yakimov, 2010).

**5.4 Breeding for thornlessness**

Creating new thorn-free cultivars is a popular direction of blackberry breeding. To date, at least 40 such cultivars have been created in the world, and these are valuable sources of genes for this trait for further selection. ‘Thornfree’, ‘Smoothstem’, ‘Black Satin’, ‘Loch Ness’, ‘Loch Tay’, ‘Chester Thornless’, ‘Triple Crown’, ‘Cacanska Bestrna’, ‘Orkan’, ‘Apache’, ‘Ouachita’ and many other cultivars are descendants of the tetraploid (2n = 4x = 28) source of the recessive gene of this trait – ‘Merton Thornless’ and can be used in breeding for thornlessness. Given that this gene is introduced into the genoplasm of relatively hardy tetraploid cultivars, they are probably the ones that should be used for breeding in the Central zone of Russia. At the early stages of ontogenesis, its carriers are identified by the absence of glandules on the germ leaves (Clark, Finn, 2011), so selection for this trait can be conducted at the very beginning of the selection process.

Two other sources of thornlessness i. e. ‘Austin Thornless’ (2n = 8x = 56) with the dominant S_f_ gene, created in England, and ‘Lincoln Logan’ (2n = 6x = 42) with the dominant S_fl_ gene, created in New Zealand, are used for breeding cultivars of the trailing type, usually with a higher level of ploidy than tetraploids. Plants with the Sf gene can have thorns on the basal part of the stem up to a height of 30 cm, in the zone of fruiting; the stems are devoid of spines. Therefore, the identification of thorn-free offspring in this case is possible only after the seedlings reach a height of more than 0.3 m (Clark, Finn, 2011). Descendants of ‘Austin Thornless’ are the thorn-free cultivars ‘Waldo’, ‘Black Perl’, ‘Nightfall’, and ‘Black Diamond’, created at the University of Oregon in the United States. Among the latest achievements of this institution is thorn-free ‘Wild Treasure’ created by crossing the ‘Waldo’ cultivar and the wild species R. ursinus (Finn et al., 2010). New thornless cultivars with the Sf l gene are ‘Columbia Star’, ’Columbia Sunrise’, and ‘Columbia Giant’ (Finn et al., 2014, 2018), which may be sources of this gene of thornlessness in further breeding. Such cultivars are more suitable for growing in the southern regions of horticulture in Russia, but with a reliable shelter for the winter, they can probably be cultivated in amateur gardens in the center of Russia.

**5.5 Breeding for resistance to pests and diseases**

Currently, the majority of blackberry cultivars grown have a high level of resistance to harmful organisms. However, the more blackberries are grown, the more often there are reports of an increased number of cases of fungal diseases and pests, with a much higher frequency than bacteria or viruses (Clark, Finn, 2011; Finn, Clark, 2012). The main blackberry breeding for resistance to diseases and pests was carried out by simple selection of healthier or uninfected plants in the seedling population (Clark, Finn, 2011). In order to understand the degree of potential danger of various pathogens, below we provide information about the most harmful of them, which are represented to varying degrees in the regions of cultivation of the discussed crop (Clark, Finn, 2011; Finn, Clark, 2012).

The most common in the world fungal diseases of blackberries are: anthracnose (Gloeosporium venetum Speg.), gray rot (Botrytis cinerea Pers.: Fr). and leptosphaeria (Leptosphaeria coniothyrium (Fuckel) Sacc.). Infection with these pathogens can vary greatly depending on the type of berries, growing locations, weather, and plant care. Orange rust (Gymnoconia peckiana (Howe) Trott.) is sometimes seen in plantings, and removal of infected plants is required. Resistance to orange rust is found in almost all eastern (erect) cultivars of the United States, with the exception of the ‘Navaho’ cultivar. For many years in the southern States of the United States, the most dangerous disease was the doubling of the rosette – cercosporellosis (Cercosporella rubi (G. Winter) Plakidas) in the production of blackberries. However, the resistance to this disease was identified, which turned out to be associated with the presence of a recessive gene of thornlessness in the blackberry genoplasm. Therefore, cultivars derived from the source of this gene – ‘Merton Thornless’ cultivars, are resistant to this disease (Clark, Finn, 2011). However, with the intense infection in the US States of Mississippi and Louisiana, the resistance was not always maintained.

In some years, Botrytis cinerea Pers.: Fr., Septoria rubi Westend, Septocyta ruborum (Lib.) Petr. and Didymella applanata (Niessl) Sacc. were observed in parts of Chile, Mexico, New Zealand, and the Western United States. Also in these areas and in areas of the UK and Europe, Peronospora sparsa Berk. can create a serious problem. Raspberry and blackberry hybrids, such as Boysenberry and Loganberry, can also be significantly affected by this disease. Powdery mildew (Podosphaera macularis Wallr.) is observed in some areas of the world, especially in Mexico. With the increase in plantings in Mexico and other areas, these diseases are becoming more economically important, and it is necessary to find sources of resistance and use them in breeding.

From bacterial diseases, root cancer (Agrobacterium tumefaciens [E.V. Smith & Townsend] Conn.) is the most common and can lead to weakening of blackberry plants and significant loss of yield. One of the main viral diseases of the Rubus genus for many years was bush dwarfism caused by Raspberry bushy dwarf virus (RBDV), but it was most pronounced in raspberry species (i. e. R. idaeus, R. parvif lorus, R. spectabilis). Tomato ringspot virus (ToRSV) and Tobacco ringspot virus (TRSV) have been identified in most regions where blackberries are produced.

All production regions have problems with blackberry pest control. The most common pests are: raspberry glassworm – Pennisetia hylaeiformis (Harris), goldenrod – Agrilus ruficollis (Fabricius), blackberry mite – Acalitus essigi Hassan., etc. Some production regions are heavily affected by pests that require active protection programs. For example, in New Zealand, the cultivars ‘Boysenberry’, ‘Marion’ and all other Rubus representatives are strongly attacked by bud moth (Heterocrossa rubophaga Dugdale and Eutorna phaulacosma Meyrick). Green vegetable beetle Nezara viridula L. and leafhopper species including Epiphyas postivittana Walker, Planotortrix exessana Walker etc. can also be a serious problem in New Zealand. In 2008, California first reported a spotted winged Drosophila (Drosophila suzukii Matsumura) native to Japan, and then it spread widely across the Western States and in China. It was recently identified in Europe. The depth of this problem has not yet been studied, but there is a danger that this pest will become the main one for blackberries. Much of the breeding effort is focused on field assessments of seedlings for pest resistance and the use of the most resistant ones in crosses (Clark, Finn, 2011; Finn, Clark, 2012).

As we can see, the main danger of diseases and pests of blackberries are for regions with a warm climate, so Central Russia may be safer for the cultivation of this crop. However, breeding for resistance to biotic environmental factors is certainly more effective with a high natural infectious background or with the use of artificial infection.

According to the observations of the authors of this article, in recent years in the Orel region, where this study is conducted, the flower-eating beetle – olenka shaggy (Epicometis hirta Poda) has spread, which begins to cause noticeable damage to early-flowering cultivars of blackberries (especially the most winter-hardy ‘Agawam’), which makes us pay special attention to this pest and cultivars that are not affected by it, for use them in breeding.

## 6 Prospects of blackberry breeding in Central Russia

The great achievements of blackberry breeding in the world, the availability of new cultivars with a complex of valuable characteristics for Russian researchers, and the work carried out on the territory of Russia to study it, allow us to consider it promising to improve the assortment of this plant not only in the South, but also in the Central part of our country. The climate of this region can contribute to the effective formation of genotypes with the necessary signs of adaptability, first of all, high frost and winter hardiness, since negative temperatures critical for the culture occur here almost every winter. At the same time, the selection process itself can be conducted in the field, without the use of artificial freezing of plants.

It is known that the majority of representatives of the subgenus Eubatus Focke belong to mesophytic plants by ecological affiliation. In the natural populations of the South of Russia, this is manifested in the fact that they are confined to fairly humid and often protected from the active sun habitats (Yuzepchuk, 1941; Grossgeim, 1952; Neronova, 1973; Ourecki, 1981). For most cultivars of this crop, similar conditions are also preferred. At the same time, in the southern zone, hot dry periods during the growing season of blackberries occur almost annually, often causing the dark berries to bake with a corresponding loss of commodity qualities and even the drying of generative organs (Semenova, Dobrenkov, 2001). In the conditions of the Orel region, where it is planned to conduct breeding research on blackberries, such periods occur much less often (2–4 times in 10 years). At the same time, they allow us to objectively assess the degree of drought tolerance of the crop in this region and, if necessary, to conduct selective selection for this indicator.

Among the other “advantages” of the Central zone of Russia, which are necessary for the cultivation of blackberries, we can mention a much lower potential danger of diseases and pests than in the South due to climate barriers that prevent their spread. Given the already high potential for resistance to these harmful factors, the crop may be very promising in this zone for organic farming, the relevance and necessity of which are recognized in many regions of the world (Doroshenko et al., 2012) and in Russia in particular (https://soz.bio/o-soyuze/). At the same time, certain vegetation periods in the middle zone are favorable for the development of fungal infections, so the identification of resistant blackberry genotypes in breeding research is also possible here.

Selection in other priority areas of blackberry breeding can be carried out in the region without any climatic or other restrictions.

## Conclusion

By 2020, VNIISPK has studied a collection of blackberries in the amount of 28 cultivars (3 cultivars were obtained from MOS VIR, 25 – from the nurseries “Divny Sad” of the Voronezh region and “Zeleny Shum” of the Orel region) of the world assortment and 18 selected and elite breeding forms obtained by the authors of the article earlier. Breeding forms were grown from seeds from open pollination of a number of cultivars sent to VNIISPK from the US University of Arkansas and the Swedish Institute of Agricultural Sciences in 1995. Elements of the technology of cultivating the varietal and selection gene pool with the use of winter shelter have been worked out. The first crosses aimed at implementing the priorities of crop breeding in the region were carried out. An annual replenishment of the blackberry collection is planned with new genetic sources of economic and useful traits, first of all, the most winter-hardy.

The analysis of world trends and experience in blackberry breeding and cultivar study, as well as the results of own researches of the crop, carried out in conditions of the Orel region, allow us to consider a promising and feasible work on improving the assortment of this plant in Central Russia.

All the priority areas and tasks of blackberry breeding identified in foreign and domestic breeding programs are relevant for this region of our country, and the most important of them is currently the creation of blackberry cultivars with high winter hardiness.

## Conflict of interest

The authors declare no conflict of interest.
